# IMPACT OF SOCIAL SUPPORT AND STRAIN ON DEPRESSIVE SYMPTOMS: MARITAL STATUS AND GENDER DIFFERENCES

**DOI:** 10.1093/geroni/igz038.3350

**Published:** 2019-11-08

**Authors:** Deborah Carr, Yeonjung Jane Lee

**Affiliations:** 1 Boston University, Boston, Massachusetts, United States; 2 University of Hawaii, Honolulu, Hawaii, United States

## Abstract

Social relationships are a well-established correlate of late-life well-being. Extensive research finds social support is associated with fewer depressive symptoms, yet few studies distinguish fine-grained types of support from spouse, children, other family and friends, nor whether these linkages differ by gender and marital status. Studies exploring coarse associations between support and well-being may conceal gender and marital status differences. We use data from two waves of the Health and Retirement Study (HRS; 2006 and 2010) to study fine-grained linkages between diverse types of relationship strain and support and depressive symptoms (CESD) among adults aged 51+. The results show that the association between support/strain and depressive symptoms varies based on the source of support. For instance, among married/partnered older adults, spousal support is negatively associated with depressive symptoms whereas friend strain is positively associated with depressive symptoms. Among widowed respondents, friend support is negatively associated with depressive symptoms. These marital status patterns differed by gender however, such that the impact of friend strain on depressive symptoms was especially large for divorced men. Our results suggest that no single form of social support (or strain) is uniformly protective (or distressing), so services and interventions to enhance late-life mental health should more fully consider older adults’ social location, including gender and marital status. For current cohorts of older adults, who have lower rates of marriage and childbearing than their predecessors, it is critically important to understand both the levels and impacts of alternative sources of support from other kin and friends.

